# Meet the Section Editor

**DOI:** 10.2174/1570159X2206231107121508

**Published:** 2023-11-07

**Authors:** Gianluca Rosso

**Affiliations:** 1 Department of Neurosciences ‘Rita Levi Montalcini’, University of Turin, Torino, Italy

   Associate Professor of Psychiatry at Department of Neurosciences ‘Rita Levi Montalcini’, University of Torino, Italy. Clinical activity at Psychiatric Unit of San Luigi Gonzaga University Hospital of Orbassano (Torino), Italy. Lecturer at University of Turin: Medicine and Surgery School and other Degree Courses, Postgraduate School of Psychiatry. The number of publications is now more than 100 publications on national and international peer reviewed journals. Research interests: mood disorders, clinical psychopharmacology, obsessive-compulsive disorder, short-term psychodynamic psychotherapy.
